# 7-Di­ethyl­amino-3-{(*E*)-4-[(*E*)-2-(pyridin-4-yl)ethen­yl]styr­yl}-2*H*-chromen-2-one

**DOI:** 10.1107/S1600536814001123

**Published:** 2014-01-22

**Authors:** Li-Ping Zhou, Ling-Liang Long

**Affiliations:** a Functional Molecular Materials Research Centre, Scientific Research Academy & School of Chemistry and Chemical Engineering, Jiangsu University, Zhenjiang, Jiangsu 212013, People’s Republic of China

## Abstract

In the title coumarin derivative, C_28_H_26_N_2_O_2_, the coumarin unit is approximately planar, with a maximum deviation of 0.048 (3) Å. The central benzene ring is oriented at dihedral angles of 30.15 (14) and 10.51 (11)°, respectively, to the pyridine ring and coumarin ring system. In the crystal, weak C—H⋯O and C—H⋯N hydrogen bonds and weak C—H⋯π inter­actions link the mol­ecules into a three-dimensional supra­molecular architecture.

## Related literature   

For applications of coumarin derivatives, see: Gong *et al.* (2012 ▶); Jones *et al.* (1985 ▶); Nemkovich *et al.* (1997 ▶); Jin *et al.* (2011 ▶); Helal *et al.* (2011 ▶).
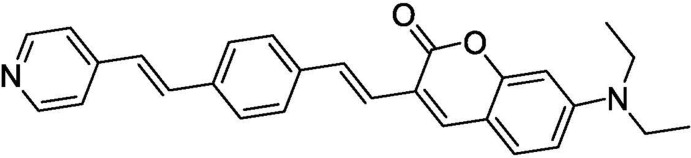


## Experimental   

### 

#### Crystal data  

C_28_H_26_N_2_O_2_
*M* *_r_* = 422.51
Monoclinic, 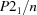

*a* = 15.511 (3) Å
*b* = 8.4745 (17) Å
*c* = 16.882 (7) Å
β = 97.73 (3)°
*V* = 2198.9 (11) Å^3^
*Z* = 4
Mo *K*α radiation
μ = 0.08 mm^−1^
*T* = 293 K
0.27 × 0.25 × 0.23 mm

#### Data collection  

Bruker APEXII CCD area-detector diffractometer
9607 measured reflections
3931 independent reflections
2993 reflections with *I* > 2σ(*I*)
*R*_int_ = 0.045

#### Refinement  

*R*[*F* ^2^ > 2σ(*F* ^2^)] = 0.077
*wR*(*F* ^2^) = 0.170
*S* = 1.13
3931 reflections
291 parameters
H-atom parameters constrained
Δρ_max_ = 0.28 e Å^−3^
Δρ_min_ = −0.21 e Å^−3^

### 

Data collection: *APEX2* (Bruker, 2008 ▶); cell refinement: *SAINT* (Bruker, 2008 ▶); data reduction: *SAINT*; program(s) used to solve structure: *SHELXTL* (Sheldrick, 2008 ▶); program(s) used to refine structure: *SHELXTL*; molecular graphics: *SHELXTL*; software used to prepare material for publication: *SHELXTL*.

## Supplementary Material

Crystal structure: contains datablock(s) I, global. DOI: 10.1107/S1600536814001123/xu5763sup1.cif

Structure factors: contains datablock(s) I. DOI: 10.1107/S1600536814001123/xu5763Isup2.hkl

Supporting information file. DOI: 10.1107/S1600536814001123/xu5763Isup3.cml

CCDC reference:

Additional supporting information:  crystallographic information; 3D view; checkCIF report

## Figures and Tables

**Table 1:** Hydrogen-bond geometry (Å, °) *Cg*2 is the centroid of the pyridine ring.

*D*—H⋯*A*	*D*—H	H⋯*A*	*D*⋯*A*	*D*—H⋯*A*
C21—H21⋯O1^i^	0.93	2.48	3.351 (4)	156
C25—H25*A*⋯N1^ii^	0.97	2.60	3.485 (4)	152
C25—H25*B*⋯O1^i^	0.97	2.56	3.433 (4)	150
C9—H9⋯*Cg*2^iii^	0.93	2.94	3.728 (4)	143

Symmetry codes: (i) 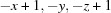; (ii) 
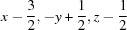
; (iii) 
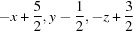
.

**Fig. 1.**
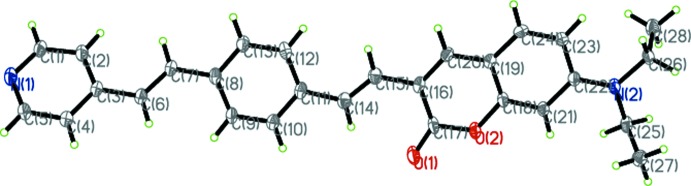
The structure of title molecule, showing the atom-numbering scheme. Displacement ellipsoids are drawn at the 30% probability level.

**Fig. 2.**
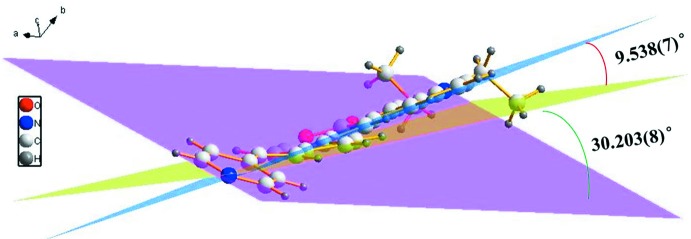
The dihedral angles (°) between adjacent planes. The pink, yellow, and blue planes represent pyridine ring, the benzene ring, and the coumarin ring.

**Fig. 3.**
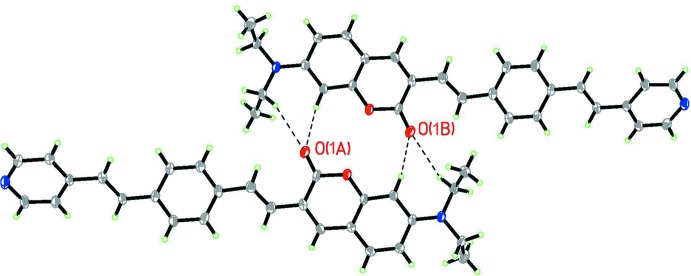
A view of the C—H···O hydrogen-bonded ring and bifurcated nature of atom O2. Hydrogen-bond interactions are shown with dashed lines.

**Fig. 4.**
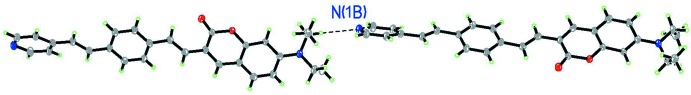
A view of the C—H···N hydrogen-bonds of atom N2. Hydrogen-bond interactions are shown with dashed lines.

**Fig. 5.**
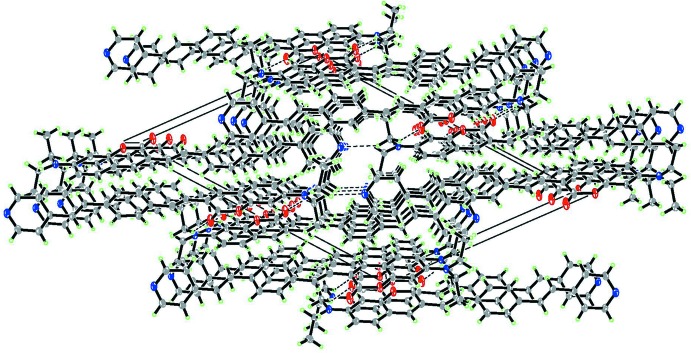
The crystal packing of title molecule, viewed along the b axis. C—H···O and C—H···N hydrogen bonds are shown as dashed lines (see Table 1 for details).

## supplementary crystallographic information

### 1. Comment

The coumarin derivatives are widely used fluorescence dye with favorable optical properties including high fluorescence quantum yield, superior photostability, and extended spectral range. These outstanding optical properties allow them to be potentially utilized in a wide range of areas such as ion sensing (Gong *et al.*, 2012), laser dyes (Jones *et al.*, 1985), nonlinear optical chromophores (Nemkovich *et al.*, 1997), fluorescent labeling of biomaterials (Jin *et al.*, 2011), and so on. In addition, previous studies have demonstrated that the optical properties of the coumarin dye could be improved by introducing conjugated group at the 3 position of the coumarin ring (Helal *et al.*, 2011). These promoted us to develop large conjugated coumarin derevatives. Herein, the synthesis and crystal structure of title molecule are presented.

The analysis of title molecule shows that it crystallizes in the monoclinic space group P 21/n with four molecules in the unit cell. In the molecule, the C17—O1 bonds, C6—C7 bonds and C14—C15 bonds show typical double-bond character (Figure 1, Table 1). The length of the C6—C7 bonds [1.333 (4) Å] compares favorably to that of the analogous C14—C15 bond [1.317 (4) Å]. On the other hand, the C17—O1 bond length [1.212 (3) Å] is shorter than that C17—O2 [1.392 (3) Å]. The coumarin ring, phenyl ring, and pyridine ring were connected by the two C—C double bonds (C6—C7 bond and C14—C15 bond), which make the three rings in good conjugation. In addition, the dihedral angles between the mean planes of the pyridine ring and the phenyl ring, the phenyl ring and the coumarin ring are 30.203 (8)°, 9.538 (7)°, respectively (Figure 2). The crystal structure of the title molecule is characterized by inter­molecular C—H···O and C—H···N hydrogen bonding (Figures 3 and 4, Table 1). The inter­molecular hydrogen-bonding scheme features a bifurcated inter­action to atom O1 and an *R*^2^_2_ (7) and *R*^2^_2_ (12) graph sets, as shown in Figure 3. The inter­molecular hydrogen-bonding scheme features an inter­action to atom N1, as shown in Figure 4. The crystal packing diagram are the fundamental linking units in the formation of a supra­molecular structure with inter­molecular C—H···O and C—H···N hydrogen-bonds, as shown in Figure 5.

### 2. Synthesis and crystallization

1N sodium hydroxide solution (1ml) was added dropwise to a solution of 7-di­ethyl­amino­coumarin-3-carbaldehyde (0.391g, 1.596mmol) and (1,4-phenyl­enebis(methyl­ene))bis­(tri­phenyl­phospho­nium) chloride (1g, 1.596mmol) in di­chloro­methane (20ml). The reaction mixture was stirred overnight at room temperature. After removal of the solvent under reduced pressure, the resulting mixture was purified by column chromatography on silica gel (di­chloro­methane: petroleum ether = 3: 7, v/v) to afford a yellow solid. Then, 1N sodium hydroxide solution (1ml) was added dropwise to the resulting yellow solid (0.5g, 0.84mmol) and isonicotinaldehyde (0.09g, 0.84mmol) in di­chloro­methane (20ml). The solution was stirred for 8 hours at room temperature. After removal of the solvent under reduced pressure, the crude product was purified by column chromatography on silica gel (di­chloro­methane: petroleum ether = 2: 3, v/v) to afford the title compound as red solid (184mg, yield 52%). Mp 265-266^o^C. The crystal appropriate for X-ray data collection was obtained from methanol- di­chloro­methane solution at room temperature after about a week.

### 3. Refinement

H atoms were positioned geometrically and refined with riding model, with *U*_iso_ = 1.2*U*_eq_ or 1.5*U*_eq_ for all H atoms. The C—H bond are 0.93 (pyridyl, aromatic), 0.96 (methyl), or 0.97Å (methyl­ene).

### Crystal data

**Table:**

C_28_H_26_N_2_O_2_	*F*(000) = 896
*M**_r_* = 422.51	*D*_x_ = 1.276 Mg m^−^^3^
Monoclinic, *P*2_1_/*n*	Mo *K*α radiation, λ = 0.71073 Å
Hall symbol: -P 2yn	Cell parameters from 9607 reflections
*a* = 15.511 (3) Å	θ = 3.5–25.5°
*b* = 8.4745 (17) Å	µ = 0.08 mm^−^^1^
*c* = 16.882 (7) Å	*T* = 293 K
β = 97.73 (3)°	Block, pink
*V* = 2198.9 (11) Å^3^	0.27 × 0.25 × 0.23 mm
*Z* = 4	

### Data collection

**Table:**

Bruker APEXII CCD area-detector diffractometer	2993 reflections with *I* > 2σ(*I*)
Radiation source: fine-focus sealed tube	*R*_int_ = 0.045
Graphite monochromator	θ_max_ = 25.2°, θ_min_ = 3.6°
phi and ω scans	*h* = −16→18
9607 measured reflections	*k* = −10→8
3931 independent reflections	*l* = −18→20

### Refinement

**Table:**

Refinement on *F*^2^	Primary atom site location: structure-invariant direct methods
Least-squares matrix: full	Secondary atom site location: difference Fourier map
*R*[*F*^2^ > 2σ(*F*^2^)] = 0.077	Hydrogen site location: inferred from neighbouring sites
*wR*(*F*^2^) = 0.170	H-atom parameters constrained
*S* = 1.13	*w* = 1/[σ^2^(*F*_o_^2^) + (0.054*P*)^2^ + 1.2201*P*] where *P* = (*F*_o_^2^ + 2*F*_c_^2^)/3
3931 reflections	(Δ/σ)_max_ = 0.004
291 parameters	Δρ_max_ = 0.28 e Å^−^^3^
0 restraints	Δρ_min_ = −0.21 e Å^−^^3^

### Special details

**Table:**

Experimental. ^1^H NMR (400MHz, CDCl_3_) δ (ppm): 8.60 (d, *J* = 6.0 Hz, 2H), 7.72 (s, 1H), 7.53 (m, 7H), 7.41(d, *J* = 16.0 Hz, 1H), 7.33 (d, *J* = 9.0 Hz, 1H), 7.19 (d, *J* = 16.4 Hz, 1H), 7.09 (d, *J* = 16.4 Hz, 1H), 6.64 (dd, *J_1_* = 2.4 Hz, *J_2_* = 8.8 Hz, 1H), 6.54 (d, *J* = 2.4 Hz, 1H), 3.48 (q, *J* = 7.2 Hz, 4H), 1.27 (t, *J* = 7.2 Hz, 6H). ESI-MS (m/z): 423.3 [M+1]^+^. Anal. calcd for C_28_H_26_N_2_O_2_: C 79.59, H 6.20, N 6.63; found C 79.34, H 6.23, N 6.61.
Geometry. All e.s.d.'s (except the e.s.d. in the dihedral angle between two l.s. planes) are estimated using the full covariance matrix. The cell e.s.d.'s are taken into account individually in the estimation of e.s.d.'s in distances, angles and torsion angles; correlations between e.s.d.'s in cell parameters are only used when they are defined by crystal symmetry. An approximate (isotropic) treatment of cell e.s.d.'s is used for estimating e.s.d.'s involving l.s. planes.
Refinement. Refinement of *F*^2^ against ALL reflections. The weighted *R*-factor *wR* and goodness of fit *S* are based on *F*^2^, conventional *R*-factors *R* are based on *F*, with *F* set to zero for negative *F*^2^. The threshold expression of *F*^2^ > σ(*F*^2^) is used only for calculating *R*-factors(gt) *etc*. and is not relevant to the choice of reflections for refinement. *R*-factors based on *F*^2^ are statistically about twice as large as those based on *F*, and *R*- factors based on ALL data will be even larger.

### Fractional atomic coordinates and isotropic or equivalent isotropic displacement parameters (Å^2^)

**Table:**

	*x*	*y*	*z*	*U*_iso_*/*U*_eq_	
N1	1.47594 (15)	0.3672 (3)	0.89342 (16)	0.0470 (7)	
N2	0.22420 (13)	0.3446 (3)	0.48620 (14)	0.0365 (6)	
O1	0.65839 (12)	0.1130 (3)	0.54400 (15)	0.0588 (7)	
O2	0.52260 (11)	0.1909 (2)	0.53258 (13)	0.0415 (5)	
C1	1.40807 (18)	0.4019 (4)	0.93132 (19)	0.0498 (8)	
H1	1.4196	0.4368	0.9839	0.060*	
C2	1.32252 (18)	0.3898 (4)	0.89825 (19)	0.0457 (8)	
H2	1.2783	0.4147	0.9283	0.055*	
C3	1.30236 (17)	0.3397 (3)	0.81916 (17)	0.0362 (7)	
C4	1.37250 (17)	0.3025 (3)	0.77916 (19)	0.0411 (7)	
H4	1.3631	0.2676	0.7265	0.049*	
C5	1.45611 (18)	0.3177 (4)	0.8182 (2)	0.0461 (8)	
H5	1.5018	0.2916	0.7901	0.055*	
C6	1.21301 (17)	0.3227 (3)	0.77934 (18)	0.0383 (7)	
H6	1.2052	0.2639	0.7324	0.046*	
C7	1.14170 (17)	0.3832 (3)	0.80373 (17)	0.0372 (7)	
H7	1.1497	0.4475	0.8487	0.045*	
C8	1.05196 (17)	0.3579 (3)	0.76635 (17)	0.0359 (7)	
C9	1.02959 (17)	0.2394 (4)	0.70924 (17)	0.0386 (7)	
H9	1.0731	0.1758	0.6934	0.046*	
C10	0.94475 (17)	0.2154 (3)	0.67630 (17)	0.0385 (7)	
H10	0.9321	0.1348	0.6392	0.046*	
C11	0.87729 (17)	0.3078 (3)	0.69667 (17)	0.0369 (7)	
C12	0.89826 (17)	0.4242 (4)	0.75531 (18)	0.0418 (8)	
H12	0.8545	0.4864	0.7716	0.050*	
C13	0.98422 (17)	0.4470 (4)	0.78924 (19)	0.0430 (7)	
H13	0.9967	0.5240	0.8283	0.052*	
C14	0.78861 (18)	0.2774 (3)	0.65598 (18)	0.0404 (7)	
H14	0.7806	0.1860	0.6254	0.048*	
C15	0.71960 (18)	0.3667 (3)	0.65861 (18)	0.0404 (7)	
H15	0.7285	0.4568	0.6901	0.048*	
C16	0.63081 (17)	0.3435 (3)	0.61866 (17)	0.0353 (7)	
C17	0.60931 (17)	0.2112 (3)	0.5649 (2)	0.0409 (7)	
C18	0.45869 (16)	0.2969 (3)	0.54637 (17)	0.0336 (7)	
C19	0.48051 (17)	0.4303 (3)	0.59359 (16)	0.0342 (7)	
C20	0.56747 (17)	0.4488 (3)	0.62915 (17)	0.0372 (7)	
H20	0.5820	0.5368	0.6611	0.045*	
C21	0.37542 (16)	0.2645 (3)	0.51058 (17)	0.0342 (7)	
H21	0.3639	0.1734	0.4803	0.041*	
C22	0.30769 (17)	0.3713 (3)	0.52046 (16)	0.0339 (7)	
C23	0.32947 (18)	0.5082 (4)	0.56701 (17)	0.0400 (7)	
H23	0.2862	0.5807	0.5743	0.048*	
C24	0.41301 (18)	0.5355 (4)	0.60136 (17)	0.0417 (7)	
H24	0.4253	0.6273	0.6309	0.050*	
C25	0.19833 (18)	0.1976 (3)	0.44438 (18)	0.0395 (7)	
H25A	0.1384	0.1744	0.4508	0.047*	
H25B	0.2342	0.1124	0.4688	0.047*	
C26	0.15537 (17)	0.4615 (4)	0.48859 (17)	0.0393 (7)	
H26A	0.1134	0.4510	0.4408	0.047*	
H26B	0.1805	0.5663	0.4884	0.047*	
C27	0.2064 (2)	0.2034 (4)	0.35653 (19)	0.0475 (8)	
H27A	0.1715	0.2883	0.3320	0.071*	
H27B	0.1867	0.1054	0.3320	0.071*	
H27C	0.2661	0.2203	0.3497	0.071*	
C28	0.1087 (2)	0.4444 (5)	0.56134 (19)	0.0551 (9)	
H28A	0.0833	0.3411	0.5617	0.083*	
H28B	0.0638	0.5227	0.5596	0.083*	
H28C	0.1495	0.4586	0.6089	0.083*	

### Atomic displacement parameters (Å^2^)

**Table:**

	*U*^11^	*U*^22^	*U*^33^	*U*^12^	*U*^13^	*U*^23^
N1	0.0280 (13)	0.0560 (17)	0.0546 (17)	−0.0014 (12)	−0.0031 (12)	0.0004 (14)
N2	0.0246 (12)	0.0407 (14)	0.0425 (14)	0.0028 (10)	−0.0018 (10)	0.0017 (11)
O1	0.0295 (11)	0.0415 (12)	0.101 (2)	0.0045 (10)	−0.0078 (12)	−0.0150 (13)
O2	0.0244 (10)	0.0315 (10)	0.0654 (14)	0.0001 (8)	−0.0053 (9)	−0.0033 (10)
C1	0.0315 (16)	0.070 (2)	0.0445 (19)	−0.0033 (16)	−0.0056 (14)	−0.0094 (17)
C2	0.0290 (15)	0.060 (2)	0.0471 (19)	−0.0009 (15)	0.0003 (13)	−0.0041 (16)
C3	0.0280 (14)	0.0391 (16)	0.0396 (17)	−0.0045 (12)	−0.0025 (12)	0.0014 (13)
C4	0.0333 (16)	0.0456 (17)	0.0434 (18)	−0.0023 (14)	0.0012 (13)	−0.0022 (15)
C5	0.0284 (15)	0.054 (2)	0.055 (2)	0.0012 (14)	0.0033 (14)	−0.0022 (17)
C6	0.0306 (15)	0.0446 (17)	0.0378 (17)	−0.0034 (13)	−0.0018 (13)	0.0003 (14)
C7	0.0282 (14)	0.0413 (17)	0.0399 (17)	−0.0038 (13)	−0.0037 (12)	0.0026 (13)
C8	0.0279 (14)	0.0412 (16)	0.0372 (17)	−0.0009 (13)	−0.0003 (12)	0.0106 (13)
C9	0.0251 (14)	0.0488 (18)	0.0407 (17)	−0.0015 (13)	−0.0006 (12)	0.0032 (14)
C10	0.0340 (15)	0.0440 (17)	0.0358 (17)	−0.0020 (14)	−0.0015 (13)	0.0039 (13)
C11	0.0305 (15)	0.0434 (17)	0.0351 (16)	−0.0067 (13)	−0.0014 (12)	0.0109 (14)
C12	0.0260 (14)	0.0458 (18)	0.054 (2)	0.0030 (13)	0.0052 (13)	0.0096 (15)
C13	0.0323 (15)	0.0440 (17)	0.0508 (19)	−0.0018 (14)	−0.0015 (14)	0.0001 (15)
C14	0.0364 (16)	0.0374 (16)	0.0467 (18)	−0.0047 (14)	0.0030 (14)	0.0050 (14)
C15	0.0360 (16)	0.0392 (17)	0.0450 (18)	−0.0033 (14)	0.0022 (14)	0.0058 (14)
C16	0.0277 (14)	0.0407 (16)	0.0357 (16)	−0.0067 (13)	−0.0022 (12)	0.0071 (13)
C17	0.0223 (14)	0.0345 (16)	0.064 (2)	0.0040 (13)	−0.0026 (14)	0.0067 (15)
C18	0.0237 (14)	0.0319 (15)	0.0438 (17)	0.0016 (12)	−0.0013 (12)	0.0048 (13)
C19	0.0298 (14)	0.0383 (16)	0.0332 (16)	−0.0022 (13)	−0.0004 (12)	0.0003 (13)
C20	0.0322 (15)	0.0419 (17)	0.0360 (16)	−0.0060 (13)	−0.0007 (12)	−0.0046 (13)
C21	0.0263 (14)	0.0293 (14)	0.0452 (17)	−0.0011 (12)	−0.0017 (12)	−0.0004 (12)
C22	0.0280 (14)	0.0425 (16)	0.0307 (16)	0.0013 (13)	0.0024 (12)	0.0027 (12)
C23	0.0328 (15)	0.0473 (18)	0.0391 (17)	0.0055 (14)	0.0012 (13)	−0.0076 (14)
C24	0.0404 (17)	0.0456 (18)	0.0382 (17)	−0.0020 (15)	0.0022 (13)	−0.0134 (14)
C25	0.0267 (14)	0.0379 (16)	0.0518 (19)	−0.0036 (13)	−0.0022 (13)	0.0041 (14)
C26	0.0275 (14)	0.0515 (18)	0.0383 (17)	0.0093 (14)	0.0022 (12)	0.0031 (14)
C27	0.0424 (17)	0.0476 (19)	0.051 (2)	−0.0014 (15)	0.0002 (15)	−0.0026 (15)
C28	0.0389 (17)	0.081 (3)	0.047 (2)	0.0071 (17)	0.0121 (15)	0.0084 (18)

### Geometric parameters (Å, º)

**Table:**

N1—C5	1.334 (4)	C13—H13	0.9300
N1—C1	1.336 (4)	C14—C15	1.317 (4)
N2—C22	1.364 (3)	C14—H14	0.9300
N2—C25	1.461 (4)	C15—C16	1.463 (4)
N2—C26	1.461 (3)	C15—H15	0.9300
O1—C17	1.212 (3)	C16—C20	1.356 (4)
O2—C18	1.381 (3)	C16—C17	1.453 (4)
O2—C17	1.392 (3)	C18—C21	1.378 (4)
C1—C2	1.372 (4)	C18—C19	1.398 (4)
C1—H1	0.9300	C19—C24	1.395 (4)
C2—C3	1.396 (4)	C19—C20	1.409 (4)
C2—H2	0.9300	C20—H20	0.9300
C3—C4	1.392 (4)	C21—C22	1.413 (4)
C3—C6	1.464 (4)	C21—H21	0.9300
C4—C5	1.379 (4)	C22—C23	1.417 (4)
C4—H4	0.9300	C23—C24	1.366 (4)
C5—H5	0.9300	C23—H23	0.9300
C6—C7	1.333 (4)	C24—H24	0.9300
C6—H6	0.9300	C25—C27	1.506 (4)
C7—C8	1.465 (4)	C25—H25A	0.9700
C7—H7	0.9300	C25—H25B	0.9700
C8—C13	1.390 (4)	C26—C28	1.514 (4)
C8—C9	1.403 (4)	C26—H26A	0.9700
C9—C10	1.373 (4)	C26—H26B	0.9700
C9—H9	0.9300	C27—H27A	0.9600
C10—C11	1.387 (4)	C27—H27B	0.9600
C10—H10	0.9300	C27—H27C	0.9600
C11—C12	1.404 (4)	C28—H28A	0.9600
C11—C14	1.475 (4)	C28—H28B	0.9600
C12—C13	1.392 (4)	C28—H28C	0.9600
C12—H12	0.9300		
			
C5—N1—C1	115.4 (3)	C20—C16—C15	120.3 (3)
C22—N2—C25	122.0 (2)	C17—C16—C15	121.0 (3)
C22—N2—C26	122.1 (2)	O1—C17—O2	114.4 (3)
C25—N2—C26	115.9 (2)	O1—C17—C16	127.7 (3)
C18—O2—C17	122.1 (2)	O2—C17—C16	117.9 (2)
N1—C1—C2	124.7 (3)	C21—C18—O2	116.6 (2)
N1—C1—H1	117.6	C21—C18—C19	123.5 (3)
C2—C1—H1	117.6	O2—C18—C19	119.9 (2)
C1—C2—C3	119.5 (3)	C24—C19—C18	116.3 (2)
C1—C2—H2	120.3	C24—C19—C20	125.2 (3)
C3—C2—H2	120.3	C18—C19—C20	118.5 (3)
C4—C3—C2	116.3 (3)	C16—C20—C19	122.7 (3)
C4—C3—C6	120.6 (3)	C16—C20—H20	118.7
C2—C3—C6	123.1 (3)	C19—C20—H20	118.7
C5—C4—C3	119.6 (3)	C18—C21—C22	119.2 (3)
C5—C4—H4	120.2	C18—C21—H21	120.4
C3—C4—H4	120.2	C22—C21—H21	120.4
N1—C5—C4	124.4 (3)	N2—C22—C21	121.4 (3)
N1—C5—H5	117.8	N2—C22—C23	120.9 (2)
C4—C5—H5	117.8	C21—C22—C23	117.7 (2)
C7—C6—C3	126.4 (3)	C24—C23—C22	121.1 (3)
C7—C6—H6	116.8	C24—C23—H23	119.5
C3—C6—H6	116.8	C22—C23—H23	119.5
C6—C7—C8	126.2 (3)	C23—C24—C19	122.2 (3)
C6—C7—H7	116.9	C23—C24—H24	118.9
C8—C7—H7	116.9	C19—C24—H24	118.9
C13—C8—C9	117.0 (3)	N2—C25—C27	113.2 (2)
C13—C8—C7	120.7 (3)	N2—C25—H25A	108.9
C9—C8—C7	122.3 (3)	C27—C25—H25A	108.9
C10—C9—C8	121.3 (3)	N2—C25—H25B	108.9
C10—C9—H9	119.4	C27—C25—H25B	108.9
C8—C9—H9	119.4	H25A—C25—H25B	107.7
C9—C10—C11	122.0 (3)	N2—C26—C28	112.9 (2)
C9—C10—H10	119.0	N2—C26—H26A	109.0
C11—C10—H10	119.0	C28—C26—H26A	109.0
C10—C11—C12	117.4 (3)	N2—C26—H26B	109.0
C10—C11—C14	118.2 (3)	C28—C26—H26B	109.0
C12—C11—C14	124.3 (3)	H26A—C26—H26B	107.8
C13—C12—C11	120.4 (3)	C25—C27—H27A	109.5
C13—C12—H12	119.8	C25—C27—H27B	109.5
C11—C12—H12	119.8	H27A—C27—H27B	109.5
C8—C13—C12	121.9 (3)	C25—C27—H27C	109.5
C8—C13—H13	119.1	H27A—C27—H27C	109.5
C12—C13—H13	119.1	H27B—C27—H27C	109.5
C15—C14—C11	126.5 (3)	C26—C28—H28A	109.5
C15—C14—H14	116.7	C26—C28—H28B	109.5
C11—C14—H14	116.7	H28A—C28—H28B	109.5
C14—C15—C16	128.8 (3)	C26—C28—H28C	109.5
C14—C15—H15	115.6	H28A—C28—H28C	109.5
C16—C15—H15	115.6	H28B—C28—H28C	109.5
C20—C16—C17	118.7 (2)		

### Hydrogen-bond geometry (Å, º)

Cg2 is the centroid of the pyridine ring.

**Table:**

*D*—H···*A*	*D*—H	H···*A*	*D*···*A*	*D*—H···*A*
C21—H21···O1^i^	0.93	2.48	3.351 (4)	156
C25—H25*A*···N1^ii^	0.97	2.60	3.485 (4)	152
C25—H25*B*···O1^i^	0.97	2.56	3.433 (4)	150
C9—H9···*Cg*2^iii^	0.93	2.94	3.728 (4)	143

Symmetry codes: (i) −*x*+1, −*y*, −*z*+1; (ii) *x*−3/2, −*y*+1/2, *z*−1/2; (iii) −*x*+5/2, *y*−1/2, −*z*+3/2.
